# To measure the amount of ocular deviation in strabismus patients with an eye-tracking virtual reality headset

**DOI:** 10.1186/s12886-021-02016-z

**Published:** 2021-06-04

**Authors:** Po-Han Yeh, Chun-Hsiu Liu, Ming-Hui Sun, Sheng-Chu Chi, Yih-Shiou Hwang

**Affiliations:** Department of Ophthalmology, Chang Gung Memorial Hospital, Chang Gung University College of Medicine, No 5, Fu-Shin Street, Kwei-Shan District, Tau-Yuan City, Taiwan

## Abstract

**Purpose:**

To investigate the accuracy of a newly developed, eye-tracking virtual reality (VR)-based ocular deviation measurement system in strabismus patients.

**Methods:**

A VR-based ocular deviation measurement system was designed to simulate the alternative prism cover test (APCT). A fixation target was made to alternate between two screens, one in front of each eye, to simulate the steps of a normal prism cover test. Patient’s eye movements were recorded by built-in eye tracking. The angle of ocular deviation was compared between the APCT and the VR-based system.

**Results:**

This study included 38 patients with strabismus. The angle of ocular deviation measured by the VR-based system and the APCT showed good to excellent correlation (intraclass correlation coefficient, ICC = 0.897 (range: 0.810–0.945)). The 95% limits of agreement was 11.32 PD. Subgroup analysis revealed a significant difference between esotropia and exotropia (*p* < 0.001). In the esotropia group, the amount of ocular deviation measured by the VR-based system was greater than that measured by the APCT (mean = 4.65 PD), while in the exotropia group, the amount of ocular deviation measured by the VR-based system was less than that of the APCT (mean = − 3.01 PD). The ICC was 0.962 (range: 0.902–0.986) in the esotropia group and 0.862 (range: 0.651–0.950) in the exotropia group. The 95% limits of agreement were 6.62 PD and 11.25 PD in the esotropia and exotropia groups, respectively.

**Conclusions:**

This study reports the first application of a consumer-grade and commercial-grade VR-based device for assessing angle of ocular deviation in strabismus patients. This device could provide measurements with near excellent correlation with the APCT. The system also provides the first step to digitize the strabismus examination, as well as the possibility for its application in telemedicine.

## Introduction

There are several methods to measure the amount of ocular deviation, including the Krimsky test, the alternative prism cover test (APCT), and the simultaneous prism cover test. The current standard to measure objective deviation is the APCT. However, there are several factors that may result in inconsistent measurements, such as the operator experience and patient cooperation. The scale of the prism jumps by 5 prism diopters (PD), when above 20 PD. Incorrectly holding the prism in the frontal plane position can lead to unreliable results [1]. It takes a distance of up to 6 m to test the distance strabismus which requires a large space in the clinic area. These factors limit measurement precision. Furthermore, the process of the examination itself is hard to record and digitalize.

Virtual reality (VR) is a 3-dimensional and computer-generated environment, which can be explored and interacted with. In the VR headset, there are 2 high-resolution screens, one for each eye, and each screen can display different scenes. The VR has fast-growing potential in various fields and is also applied to rehabilitation and visual-related research [2, 3]. Currently, there are some commercial VR headsets with built-in eye tracking, which mostly utilize the signal detected by the infrared camera. To measure eye movement, the infrared camera detects the position of the central pupil in relation to the corneal reflex [4]. To our knowledge, there are few devices which measure ocular deviation by a VR-based system, and exact correlation with the APCT is unknown [5]. In our research, we attempted to simulate the prism cover test in an environment of VR, and to compare measurement correlations with the traditional APCT in strabismus patients.

## Methods

### Participants

This prospective study was conducted between September 2019 and June 2020 at Chang Gung Memorial Hospital (Taoyuan, Taiwan). Research protocols were approved by the institutional review board and also conform to the tenets of the Declaration of Helsinki. Patients with strabismus aged from 13 to 65 years were recruited from our ophthalmology clinic. All types of strabismus were included, whether the deviation was comitant, incomitant, horizontal or vertical. Patients with spherical equivalent refractive errors of greater than two diopters were excluded to prevent the effect of accommodation. Patients younger than 12 years of age were excluded according to the product safety regulations. Patients older than 65 years of age were also excluded due to poor cooperation.

Patients with uncorrected vision of less than 0.05 in either eye or an ocular disorder interfering with the eye-tracking system, such as ptosis, irregular pupil and cornea opacity, or those unable to cooperate were also excluded.

### Strabismus measurements

Enrolled patients received a strabismus examination with the APCT in clinic by a single physician (Dr. Liu). During the same visit after testing with the APCT, the patient was brought to another room, where the VR device was located. The examination with the VR device was performed by a different physician (Dr. Yeh) who was blind to clinical measurement results. Measurement results included the deviation-type of strabismus (e.g. comitant or not); the direction of deviation (i.e. esotropia, exotropia, hypertropia); and the angle of deviation, all of which were recorded separately.

### Device

We selected the VIVE Pro Eye (HTC Corporation, New Taipei City, Taiwan) as our VR-based system. It is a commercial VR headset with built-in eye tracking. The VR environment of this research was developed by Unity and simulated using VIVE Pro Eye. The VIVE Pro Eye can provide resolution of 1440 × 1600 pixels per eye with a refresh rate of 90 Hz and a field of view of 110°. The inter-pupillary distance of the device was adjustable (60.7–73.5 mm). The eye-trackers built into the device have a 120 Hz gaze data output frequency. The accuracy was 0.5°–1.1°. The eye-tracking system provides the direction of line of sight of each eye in addition to the direction of line of sight in 3D.

### VR environment

**Fig. 1:**
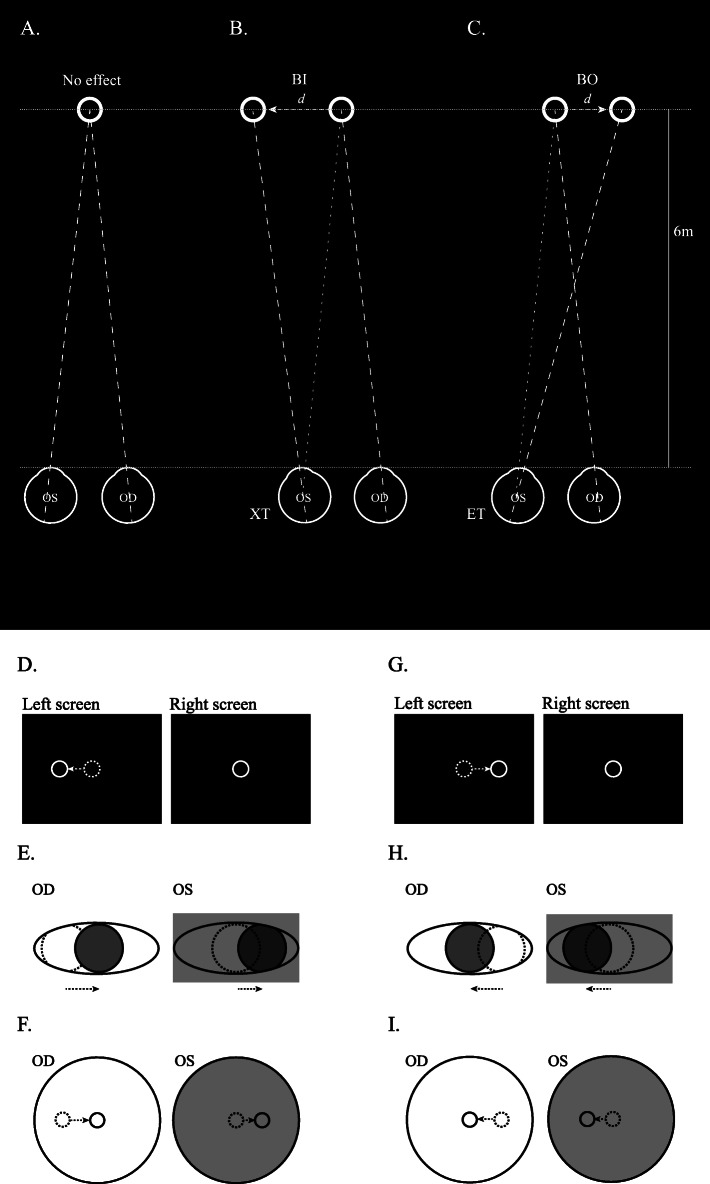
**A**-**C**, the environment of virtual reality; **D**-**F**, virtual reality test in exotropic patients; G-I, virtual reality test in esotropic patients. (**A**) In orthotropic status, the targets of both eyes are superimposed without virtual prism effect. (**B**) In exotropic status, the target of the exotropic eye (left eye in this figure) is moved temporally to simulate a virtual prism base-in (BI) effect. (**C**) In esotropic status, the target of the esotropic eye (left eye in this figure) is moved nasally to simulate a virtual prism base-out (BO) effect. In B and C, the virtual distance between 2 targets is *d* (cm), and the distance between the eye and the virtual targets is 6 m. Thus, the virtual prism effect is *d*/6 (PD). The screens that patients see on the virtual reality device with each eye (**D**, **G**), the movement of the eyes (**E**, **H**), and the real-time result by the eye- tracking system (**F**, **I**) are demonstrated separately. The blackening of one screen simulating occlusion in the cover test is presented with gray background in Figs. **E**, **F**, **H** and **I** (left eye in these examples). The dotted circle is the position of the eye before occlusion, and the solid circle is in the position after occlusion. (**D**-**F**) In exotropic patients, when the screen of the left eye is blackened, the right eye moved nasally to track the target. A leftward movement of both eyes was able to be detected by the eye-tracking system. Next, a virtual prism base-in effect as shown in Fig. B was introduced to move the target of the left eye temporally. The operator then again blackened the screens until both eyes remained still. The final amount of introduced virtual prism base-in effect was then calculated to the angle of the ocular deviation (virtual prism effect *d*/6 (PD) as shown in Fig. **B**). (**G**-**I**) In esotropic patients, when the screen of the left eye is blackened, the right eye moved temporally to track the target. A rightward movement of both eyes was able to be detected by the eye- tracking system. Next, a virtual prism base-out effect as shown in Fig. **C** was introduced to move the target of the left eye nasally. The operator then again blackened the screens until both eyes remained still. The final amount of introduced virtual prism base-in effect was then calculated to the angle of the ocular deviation (virtual prism effect *d*/6 (PD) as shown in Fig. **C**). BI, base-in; BO, base-out; XT, exotropia; ET, esotropia; PD, prism diopter; m, meters

The VR environment was designed as a black background. The virtual target was a white circle, which was set at 6 virtual meters away from the patient. The setting of a 6 m distance in a virtual environment is to simulate the 6-m distance between the target and both eyes. The diameter of the target was 20 cm, a size which is easy for a patient with uncorrected vision to see. The operator was able to move the target in any direction within a plane of 6 m, and the distance of movement could be calculated into the prism effect (Fig. 1A-C). Patients were able to see each target in each eye on the screens. To simulate the prism effect, the target was moved on the screens. For example, to simulate the effect of base-in prism in exotropia, the target image was moved to the temporal side from the neutral position, and to simulate the effect of base-out prism in esotropia, the target image was moved to the nasal side from the neutral position (Fig. 1D-G). During the examination, the operator was able to blacken each screen to simulate the occluder used in the cover test. Patients’ eye movements were observed by eye-tracking; this provided the direction of real-time gaze (Fig. 1E, F, H, I). Through eye-tracking, the operator was able to discern whether the eye moved or not, and the amount of deviation was measured by calculating the change of direction of the eye. Because there might be inaccuracy in determining gaze direction by eye-tracking, the final measurement result depended on the virtual distance between the virtual targets in each eye [6].

### Experiment procedure

- Step I: The patient put on the VR headset after the corrected inter-pupillary distance was checked by the operator. The patient was asked to open both eyes and stare at the virtual targets in the VR environment.
- Step II: The operator blackened each screen alternately and observed ocular movements by eye-tracking. This step was to simulate the alternative cover test. The direction of eye deviation was recorded by observing ocular movements. For example, left deviation would be observed on an exotropic patient when the left screen was blackened. Right deviation would be seen on an esotropic patient when the left screen was blackened. This step was to simulate the cover test (Fig. 1E, H).
- Step III: The operator fixed the target in front of one eye and let the target of the other eye move to the direction of deviated vision. This was to simulate the prism effect (Fig. 1D, G).
- Step IV: The operator blackened each screen alternatingly to observe ocular movements by eye-tracking. This step was to simulate the alternative cover test again to observe ocular movements. If the patient’s eyes moved, the operator would observe the movement and change the target position again. If the patient’s eyes remained still, the operator would record the target as the correct position in each eye. The degree of ocular deviation was calculated by the virtual position of the two targets of each eye (Fig. 1B, C). The virtual distance between the patient and target was 6 m. If the deviated distance of the target was *d* (cm), then the virtual prism diopter was *d*/6 PD.

In cases with combined vertical and horizontal deviation, the oblique virtual distance (*d*) between the two targets was on the plane of 6 m. The oblique virtual distance was represented as the hypotenuse of a right triangle. The horizontal and vertical legs of the right triangle represented horizontal and vertical deviation, respectively. Therefore, to calculate the total amount of vertical and horizontal distance, we took the square root of the sum of the squares. In patients with incomitant strabismus, we placed the simulated prism over the affected eye of incomitant strabismus to check the primary deviation. The amount of ocular deviation was determined by primary deviation.

### Statistical analysis

The agreement between the APCT and VR was determined using the intra-class correlation coefficient (ICC) with 95% confidence intervals. The ICC and the 95% confidence intervals were calculated using the SPSS statistical package version 26 (SPSS Inc., Chicago, IL), based on 2-way mixed effects, consistency, and multiple measurements. The Bland and Altman plot was used for determining the difference between the APCT and VR [7]. The 95% limits of agreement between the APCT and VR measurements was calculated to provide an estimate of the disagreement between the APCT and the VR measurements. The 95% limits of agreement was calculated by multiplying the within-subject standard deviation of the individual measures by 1.96.

The difference between the APCT and VR among the subgroups was calculated with independent samples t-test. A *p*-value< 0.05 was considered to be statistically significant.

## Results

Forty subjects were included in the present study. Two subjects with very large horizontal deviation (> 50 PD) were excluded as outliers, and will be discussed separately. As a result, 38 subjects were included in the final data analysis. The demographic characteristics of the study subjects are listed in Table 1. Mean age was 39.4 ± 16.0 (standard deviation) years (range, 14–65 years); 21 (55.3%) were female. The mean VA by logMAR was 0.619 ± 0.482 (range, 0.05–1.0 by Snellen VA). The spherical equivalent ranged from − 9.5 D (diopter) to + 2.0 D, with an average of − 3.56D ± 2.95 D. None of them wore prism for the strabismus. There were 16 subjects with exotropia, 18 with esotropia, and 4 with vertical deviation. Among these, 17 (44.7%) had incomitant strabismus. The causes of incomitant strabismus included thyroid eye disease, idiopathic myositis, stroke, cranial nerve (CN) 3, 4, 6 palsy, brain tumor, and traumatic orbital wall fracture. The mean deviation for the APCT was 25.1 ± 13.5 PD.

**Table 1:** Demographic and clinical data of patients

Parameter	Patients (***n*** = 38)
**Age (year)**	39.4 ± 16.0 (14 to 65)^a^
**Sex**	
**Female, No (%)**	21 (55.3)
**Male, No (%)**	17 (44.7)
**Visual acuity (logMAR)**	0.619 ± 0.482 (0.0 to 1.3)^a^
**Spherical equivalent (diopters)**	−3.56 ± 2.95 (−9.5 to + 2.0)^a^
**Amount of deviation by APCT (PD)**	25.1 ± 13.5 (6.0 to 51.0)^a^
**Type of strabismus**	
**XT, No (%)**	16 (42.1)
**ET, No (%)**	18 (47.4)
**Vertical, No (%)**	4 (10.5)
**Comitant, No (%)**	21 (55.3)
**Incomitant, No (%)**	17 (44.7)

*APCT* alternative prism cover test, *XT* exotropia, *ET* esotropia, *PD* prism diopter

^a^ Data are the mean ± standard deviation (minimum to maximum)

**Fig. 2:**
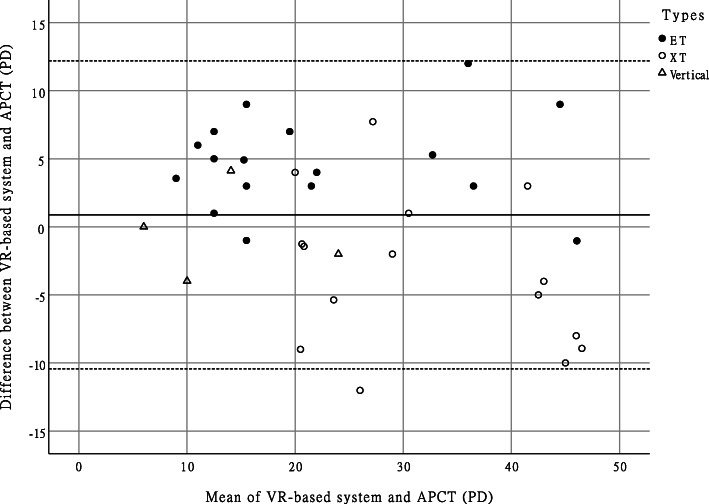
Bland-Altman Plot of the difference between the VR-based system and the APCT vs. the mean of the VR-based system and the APCT. Upper and lower dotted lines represent the 95% limits of agreement. The half-width of the 95% limits of agreement between the 2 measurements was 11.32 PD. The solid line represents the mean difference, which was 0.88 PD. VR, virtual reality; APCT, alternative prism cover test; XT, exotropia; ET, esotropia; PD, prism diopter

There were no significant measurement differences between the APCT and VR among any of the subjects (mean difference: 0.88 ± 5.77 PD, *p* = 0.352). The ICC comparing the APCT and VR was 0.897 (0.810–0.945, *p* < 0.001). In the Bland & Altman table, the 95% limits of agreement between these 2 tests was 11.32 PD. (Fig. 2).

For subgroup analysis (Table 2), the mean difference for the APCT and VR did not show a statistically significant differences between comitant and incomitant subgroups (mean differences = 1.16 PD and 0.54 PD, respectively, *p* = 0.746). However, the mean difference between for these two methods between esotropia and exotropia subgroups did demonstrate a significant difference (4.65 PD and − 3.01 PD, respectively, *p* < 0.001). Furthermore, the ICC comparing the APCT and VR in the esotropia subgroup was 0.962 (0.902–0.986, *p* < 0.001). The ICC between the APCT and VR in the exotropia subgroup was 0.862 (0.651–0.950, *p* < 0.001). The 95% limits of agreement between the APCT and VR were 6.62 PD and 11.27 PD in the esotropia and exotropia subgroups, respectively.

**Table 2:** The extent of concordance between the APCT and VR for measurement of the strabismic angle

	VR-APCT (PD)^a^	*P* value	ICC (95%CI)	95% Limits of agreement (PD)
Total (*n* = 38)	0.88 ± 5.77	0.352	0.897 (0.810–0.945)	11.32
Subgroup analysis 1				
XT (*n* = 16)	−3.01 ± 5.75	< 0.001^b^	0.862 (0.651–0.950)	11.27
ET (*n* = 18)	4.65 ± 3.38		0.962 (0.902–0.986)	6.62
Subgroup analysis 2				
Comitant (*n* = 21)	1.16 ± 6.32	0.746	0.864 (0.695–0.943)	12.39
Incomitant (*n* = 17)	0.54 ± 5.19		0.890 (0.724–0.959)	10.17

*VR* virtual reality, *APCT* alternative prism cover test, *ICC* intra-class correlation coefficient, *XT* exotropia, *ET* esotropia, *PD* prism diopter

^a^Data are mean ± standard deviation

^b^Statistically significant by the independent Samples t test among XT and ET subgroups

## Discussion

In this study, we used a consumer-grade and commercial VR headset with built-in eye-tracking to measure ocular deviation. To the best of our knowledge, this is the first study to demonstrate a correlation comparing VR-based measurement using a consumer-grade and commercial VR system in strabismus patients with that of the traditional APCT.

The present study compares the correlation between the APCT and a VR-based system in strabismus patients. Previous study has revealed that the inter-observer ICC for the APCT was 0.96 in esotropia patients with CN 6 palsy; this signifies a high concordance using the APCT among the operators [8]. Another study comparing the Krimsky test and the APCT showed that the ICC between both methods was 0.738 in exotropia cases and 0.651 in esotropia cases. Although the Krimsky test is also frequently used in the clinical setting, concordance between the Krimsky test and the APCT is reportedly not good [9]. Our study demonstrates the ICC between the APCT and VR is good-to-excellent (0.897) for all deviation types, and is even better in the esotropia subgroup (0.962). Therefore, measurements using the VR-based system has near-excellent correlation with APCT measurements [10].

Our study reveals that the current VR-based system is able to provide 11.32 PD for the 95% limits of agreement regardless of deviation type. Furthermore, our VR-based system is able to provide 6.62 PD for the 95% limits of agreement in the esotropia subgroup. In contrast to our result, a previous study showed that the 95% inter-observer limits of agreement for the alternative prism cover test was 10.2 PD in patients with CN 6 palsy [8]. Therefore, our VR-based system shows potential in practice for measuring the degree of deviation in strabismus patients with acceptable agreement.

Subgroup analyses revealed that the degree of deviation measured by VR was less than that seen with the APCT in the exotropia subgroup. On the other hand, this measurement by VR was more than that seen with the APCT in the esotropia subgroup. This means there is a tendency to overestimate deviation in esotropic patients, and to underestimate deviation in exotropic patients. In other words, the measurement by VR tends to reflect an esotropic shift in both XT and ET cases. The cause of this esotropic shift is not clearly understood. One possible reason may be due to accommodation. In the event that the screen of the VR-based system is not placed far enough away (> 6 m), accommodation may occur. Although there is no publicly available documentation regarding the focal distance of VIVE Pro Eye, a response to our inquiry to the company revealed the focal distance of VIVE Pro Eye to be about 75 cm. Accommodation in response to this focal distance may lead to significant convergence and thereby produce an esotropic shift. In addition, previous studies have shown that either a binocular or a VR device can create an oculomotor conflict, which would cause an increase, not only in AC/A ratio, but also in near and far phorias [11, 12]. By increasing the AC/A ratio and causing an esophoric shift, the oculomotor conflict would also result in overestimating esotropia and underestimating exotropia.

Although the difference between the two is small, a comparison between VR and the APCT reveals a large standard deviation (0.88 ± 5.77 PD). While the exact cause for such a large standard deviation was not clearly established, we speculate there were 2 reasons. First, larger differences occurred in cases with large degrees of strabismus, especially exotropic deviation. Such large differences may have caused the large standard deviation. Second, in the standard loose plastic prism set, are 5-PD increments from 20 to 50 PD. The 5-PD increments may also have caused the large standard deviation. Furthermore, our study reveals that the standard deviation in the exotropic subgroup is much higher than in the esotropic subgroup, and the ICC is lower in the exotropic subgroup compared to that in esotropic subgroup. A higher inconsistent rate in intermittent exotropia was also noted by other researchers. Specifically, they reported that more than half of patients had variable degrees of exotropia during preoperative measurement. The largest difference between measurements was as high as 20 PD. [13] Because intermittent exotropia showed variable extent and inconsistent results, the intrinsic variability of this condition may cause less consistency in the exotropic group in our research.

There were 2 outliers with horizontal deviations greater than 50 PD. Since 2 prisms are required to perform APCT (one in front of each eye), such cases can produce measurement errors. We would like to discuss the two cases involving very large deviations (> 50 PD), separately. The first one was in a 61-year-old male with incomitant esotropia due to a brainstem tumor. The degree of deviation was 68 PD as measured by the APCT and 73 PD by the VR based system. The second case was an 18-year-old female with comitant esotropia. The degree of deviation was 100 PD by the APCT and 114 PD by the VR based system. Both cases revealed similar measurement results with the two methods, even though deviation was very large.

The VR device with eye-tracking system offers several advantages. First, neither a prism nor an occluder is not necessary during the test. Therefore, the operative error of the prism is avoided during measurement. Second, it can provide several kinds of target images during the test. For example, using a dynamic, rather than, a static target can allow the patient to better focus on it; also, a larger sized target is easier to see [14]. Third, because the virtual prism effect is made by actually moving the target, there would be no prism step in the VR environment, even in cases with a large deviation. In our research, the largest ocular deviation was up to 114 PD. Given that the largest scale of plastic prism is 50 PD, any deviation larger than 100 PD, is difficult to measure by the APCT. Fourth, the occluder is simulated with a blackened screen, so we are still able to monitor the eye movements, even when covered. The degree of ocular movement can be quantified by the eye-tracking system. Although we did not use the data by quantified the eye-tracking system to calculate final results due to accuracy, we did use the data to estimate degree of deviation, allowing for faster examination. Last but not least, we obtained measurements using only the computer and VR device. In short, examinations do not need to be performed face to face with the patients. The VR system not only digitalizes the traditional alternative prism cover test, but also can be applied to telemedicine. In the future, such a system has the potential to provide automated measurements of the angle of strabismus.

Although the correlation of our VR-based system to the APCT is nearly excellent, there are still some possible sources of error, such as poor fitting of the headset and incorrect setting of the inter-pupillary distance. Although the headset provides a function for level correction, a poorly-fitting headset may affect measurement of vertical strabismus, if the device is not worn perfectly horizontally. Although the VR headset may still fit, an incorrect setting of the inter-pupillary distance may, nevertheless, affect the measurement of horizontal strabismus. An overestimated inter-pupillary distance make the patient’s eyes appear to be relatively esotropic, and an underestimated inter-pupillary distance may make the patient’s eyes appear to be relatively exotropic. However, to reduce this error, the VIVE Pro Eye provides for an adjustable range to fit the patients’ inter-pupillary distance. The VIVE Pro Eye does not provide for a focal distance greater than 6 m; therefore, accommodation may occur as described above. To address this situation, an alternative VR headset should be considered, in order to provide adequate focal distance. Unfortunately, no such VR headset is currently available.

There are some limitations in our study. The sample size was relatively small. We did not correct for patient’s refractive error, because the frame of glasses might interfere with the eye-tracking system. In addition, the prism effect associated with eyeglasses can lead to measurement error, particularly when the center of the lens does not align with the center of the pupil; this may occur especially in the presence of ocular deviation. Additionally, we only compared the measurement by the VR-based system with that of the APCT, and we did not determine intra-observer and inter-observer data from measurements with the VR-based system. We included all types of strabismus, such as comitant and incomitant strabismus, which might also affect measurements. However, in order to simulate the APCT in a real clinic situation, we only measured the ocular deviation in the primary position. It is reasonable to use VR for all types of deviation. In fact, there was no significant difference between comitant and incomitant subgroups in our study. Whether the deviation is comitant or not may not affect the results of the VR-based system. Furthermore, the amount of deviation observed by VR was actually assessed by a physician, not by software, so it may also be affected by the individual physician’s experience level. However, the main goal of our research was to compare the APCT to the VR device with eye-tracking. We, therefore, view the VR-based system as one type of resource, not as an instrument to replace physicians.

## Conclusion

This study reports the first application of a consumer-grade and commercial VR headset with built-in eye-tracking to assess angle of deviation in strabismus patients. Using the VR headset with built-in eye-tracking provides a feasible way to measure the angle of deviation without a prism or occluder. It provides near-excellent ICC with the APCT, and it is also a first step towards digitization of the strabismus examination.

## Data Availability

The datasets used and/or analyzed during the current study are available from the corresponding author on reasonable request.

## Abbreviations

VR: virtual reality
APCT: alternative prism cover test
ICC: intra-class correlation coefficient
PD: prism diopter
XT: exotropia
ET: esotropia
BI: base-in
BO: base-out

## References

[1] Thompson JT, Guyton DL (1983). Ophthalmic prisms. Measurement errors and how to minimize them. Ophthalmology.

[2] Maggio MG, Russo M, Cuzzola MF, Destro M, La Rosa G, Molonia F, Bramanti P, Lombardo G, De Luca R, Calabro RS (2019). Virtual reality in multiple sclerosis rehabilitation: a review on cognitive and motor outcomes. J Clin Neurosci.

[3] Iskander J, Hossny M, Nahavandi S (2019). Using biomechanics to investigate the effect of VR on eye vergence system. Appl Ergon.

[4] Brunyé TT, Drew T, Weaver DL, Elmore JG (2019). A review of eye tracking for understanding and improving diagnostic interpretation. Cogn Res Princ Implic.

[5] Miao Y, Jeon JY, Park G, Park SW, Heo H (2020). Virtual reality-based measurement of ocular deviation in strabismus. Comput Methods Prog Biomed.

[6] Guestrin ED, Eizenman M (2006). General theory of remote gaze estimation using the pupil center and corneal reflections. IEEE Trans Biomed Eng.

[7] Bland JM, Altman DG (1986). Statistical methods for assessing agreement between two methods of clinical measurement. Lancet.

[8] Holmes JM, Leske DA, Hohberger GG (2008). Defining real change in prism-cover test measurements. Am J Ophthalmol.

[9] Joo KS, Koo H, Moon NJ (2013). Measurement of strabismic angle using the distance Krimsky test. Korean journal of ophthalmology : KJO.

[10] Koo TK, Li MY (2016). A guideline of selecting and reporting Intraclass correlation coefficients for reliability research. Journal of chiropractic medicine.

[11] Neveu P, Priot AE, Plantier J, Roumes C (2010). Short exposure to telestereoscope affects the oculomotor system. Ophthalmic Physiol Opt.

[12] Mon-Williams M, Wann JP, Rushton S (1993). Binocular vision in a virtual world: visual deficits following the wearing of a head-mounted display. Ophthalmic Physiol Opt.

[13] Khazaei S, Mansori K (2018). Variability of preoperative measurements in intermittent exotropia and its effect on surgical outcome. J Aapos.

[14] Yang HK, Hwang JM (2011). The effect of target size and accommodation on the distant angle of deviation in intermittent exotropia. Am J Ophthalmol.

